# The Effects of Melatonin on the Descending Pain Inhibitory System and Neural Plasticity Markers in Breast Cancer Patients Receiving Chemotherapy: Randomized, Double-Blinded, Placebo-Controlled Trial

**DOI:** 10.3389/fphar.2019.01382

**Published:** 2019-11-22

**Authors:** Ana Claudia Souza Palmer, Andressa Souza, Vinicius Souza dos Santos, José Antônio Crespo Cavalheiro, Fernando Schuh, Angela Erguy Zucatto, Jorge Villanova Biazus, Iraci Lucena Da S. Torres, Felipe Fregni, Wolnei Caumo

**Affiliations:** ^1^Post-graduate Program in Pharmacology and Therapeutics, Department of Pharmacology, Universidade Federal do Rio Grande do Sul (UFRGS), Porto Alegre, Brazil; ^2^Postgraduate Program in Health and Human Development, La Salle University Center, Canoas, Brazil; ^3^Post-graduate Program in Medical Sciences, School of Medicine, Universidade Federal do Rio Grande do Sul (UFRGS), Porto Alegre, Brazil; ^4^Division of Breast Surgery, Hospital de Clinicas de Porto Alegre (HCPA), Postgraduate Program in Gynecology and Obstetrics, Universidade Federal do Rio Grande do Sul (UFRGS), Porto Alegre, Brazil; ^5^Pharmacology Department, Instituto de Ciências Básicas da Saúde, Universidade Federal do Rio Grande do Sul (UFRGS), Porto Alegre, Brazil; ^6^Spaulding Neuromodulation Center, Spaulding Rehabilitation Hospital, Harvard Medical School, Boston, MA, United States; ^7^Anesthesiology, Pain and Palliative Care Service, Hospital de Clínicas de Porto Alegre (HCPA), Department of Surgery, School of Medicine, Universidade Federal do Rio Grande do Sul (UFRGS), Porto Alegre, Brazil

**Keywords:** breast cancer, chemotherapy, melatonin, brain-derived neurotrophic factor, S100B protein, sleep quality

## Abstract

**Background:** Adjuvant chemotherapy for breast cancer (ACBC) has been associated with fatigue, pain, depressive symptoms, and disturbed sleep. And, previous studies in non-cancer patients showed that melatonin could improve the descending pain modulatory system (DPMS). We tested the hypothesis that melatonin use before and during the first cycle of ACBC is better than placebo at improving the DPMS function assessed by changes in the 0–10 Numerical Pain Scale (NPS) during the conditioned pain modulating task (CPM-task) (primary outcome). The effects of melatonin were evaluated in the following secondary endpoints: heat pain threshold (HPT), heat pain tolerance (HPTo), and neuroplasticity state assessed by serum brain-derived neurotrophic factor (BDNF), tropomyosin kinase receptor B, and S100B-protein and whether melatonin’s effects on pain and neuroplasticity state are due more so to its impact on sleep quality.

**Methods:** Thirty-six women, ages 18 to 75 years old, scheduled for their first cycle of ACBC were randomized to receive 20mg of oral melatonin (n = 18) or placebo (n = 18). The effect of treatment on the outcomes was analyzed by delta (Δ)-values (from pre to treatment end).

**Results:** Multivariate analyses of covariance revealed that melatonin improved the function of the DPMS. The Δ-mean (SD) on the NPS (0–10) during the CPM-task in the placebo group was −1.91 [−1.81 (1.67) vs. −0.1 (1.61)], and in the melatonin group was −3.5 [−0.94 (1.61) vs. −2.29 (1.61)], and the mean difference (md) between treatment groups was 1.59 [(95% CI, 0.50 to 2.68). Melatonin’s effect increased the HPTo and HPT while reducing the (Δ)-means of the serum neuroplasticity marker in placebo vs. melatonin. The Δ-BDNF is 1.87 (7.17) vs. −20.44 (17.17), respectively, and the md = 22.31 [(95% CI = 13.40 to 31.22)]; TrKB md = 0.61 [0.46 (0.17) vs. −0.15 (0.18); 95% CI = 0.49 to 0.73)] and S00B-protein md = −8.27[(2.89 (11.18) vs. −11.16 (9.75); 95% CI = −15.38 to −1.16)]. However, melatonin’s effect on pain and the neuroplastic state are not due to its effect on sleep quality.

**Conclusions:** These results suggest that oral melatonin, together with the first ACBC counteracts the dysfunction in the inhibitory DPMS and improves pain perception measures. Also, it shows that changes in the neuroplasticity state mediate the impact of melatonin on pain.

**Clinical Trial Registration:** www.ClinicalTrials.gov, identifier NCT03205033.

## Introduction

Chemotherapy treatment for breast cancer has been associated with fatigue, pain, depressive symptoms, and disturbed sleep (Given et al., 2001; Eversley et al., 2005; Lévi, 2006; Rief et al., 2011). Even in healthy women, sleep deprivation produces a significant decline in descending pain-inhibitory functions [i.e. a loss of diffuse noxious inhibitory controls], and an increase in spontaneous painful symptoms (Smith et al., 2007). Indeed, these previous findings affirm that poor sleep quality is a risk factor for exacerbation of chronic pain (Affleck et al., 1996; Kaila-Kangas et al., 2006). Accordingly, previous studies showed that melatonin can improve both sleep quality and pain measures (i.e. endometriosis and fibromyalgia) (Schwertner et al., 2013; de Zanette et al., 2014). Also, it optimizes the descending pain modulatory system (DPMS) (Zanette et al., 2014).

Additionally, experimental models show that the anti-inflammatory properties of melatonin reduced nuclear factor κB (NF-κB) activity, a transcription factor found within neurons and glial cells (Lezoualc and Sparapani, 1998; Kaltschmidt et al., 2005; Jumnongprakhon et al., 2015). NF-κB regulates cellular processes such as migration, maturation, plasticity and synaptic communication and it is constitutively activated in glutamatergic neurons (Grilli and Memo, 1999; Malek et al., 2007). *In vitro* studies revealed that melatonin resists microglial cytotoxicity by suppressing apoptosis and inhibiting the activity of NF-κB (Jang et al., 2005). Also, such activated cytokines may induce the secretion of neurotrophins such as brain-derived neurotrophic factor (BDNF) and S100β-protein (Lévi, 2006; Bower et al., 2011).

BDNF has been positively correlated with the potency of the DPMS (Botelho et al., 2016). Also, it modulates excitatory and inhibitory transmission through the activation of glutamatergic NMDA receptors and inhibitory GABA receptors (Whitehead et al., 2004). The primary BDNF receptor, tropomyosin kinase B (TrkB), can be a predictive marker of poor clinicopathological prognosis in breast cancer patients (Zhang et al., 2016), while preclinical studies have shown that inhibiting TrkB leads to favorable effects in neuropathic pain (Wang et al., 2009). A positive correlation between BDNF and central sensitization (CS) has been shown in humans and carries a central role in the pathophysiology of chronic pain (Caumo et al., 2017).

Overall, this set of evidence suggests that the benefits of neuroprotective effects of melatonin can counteract the neurotoxic effects induced by adjuvant chemotherapy for breast cancer (ACBC) on neuroplastic mechanisms involved in the pathophysiology of pain associated with chemotherapy. Thus, we tested the hypothesis that supplementing patients with melatonin before and during the first cycle of ACBC is better than placebo. We tested the hypothesis that melatonin use before and during the first cycle of ACBC is better than placebo to improve the DPMS function assessed by changes on the 0–10 Numerical Pain Scale (NPS) during the conditioned pain modulating (CPM) task (primary outcome). Melatonin’s effects were evaluated in the following secondary endpoints: heat pain threshold (HPT), heat pain tolerance (HPTo), and the neuroplasticity state assessed by serum BDNF, TrkB, S100B-protein, and whether melatonin’s effects on pain and the neuroplasticity are due more so to its impact on sleep quality.

## Materials and Methods

### Study Design and Eligibility

**Figure 1:**
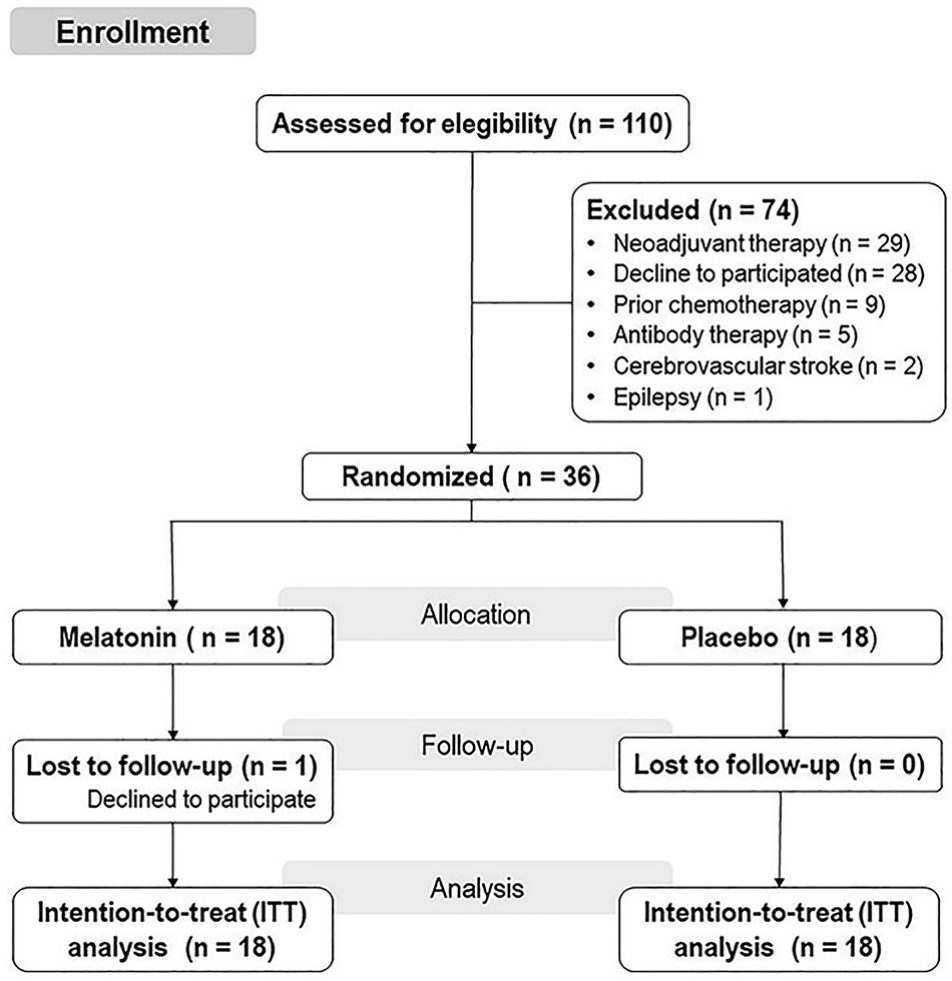
Flowchart of the study.

This randomized, double-blinded, placebo-controlled trial was approved by the Institutional Review Board of Hospital de Clínicas of Porto Alegre (IRB HCPA/Approval number: 14-0701), and it was registered on http://www.clinicaltrials.gov/ (No NCT03205033 Study start: January 2016, End date: April 2017) before inclusion of the first patient. We obtained oral and written informed consent from all patients before participating in this study. The identified data related to interventions and primary outcomes will be available upon request to interested to Caumo W (Wcaumo@hcpa.edu.br) with no time restriction. Flow of this study is presented in **Figure 1**.

### Participants

Patients were selected from the Mastology and Oncology Service at HCPA, a public tertiary teaching Medical School. Females aged 18 to 75 years with the capacity to read and write were selected. *Inclusion criteria*: females scheduled for their first cycle of ACBC one month following lumpectomy or mastectomy. *Exclusion criteria*: patients with previous chemotherapy, patients planned for neoadjuvant chemotherapy, or those with prior or other concurrent malignancies. Also excluded were patients with a history of melatonin allergy, sleep apnea, diabetes, autoimmune disease (i.e. systemic lupus erythematosus, type I diabetes, rheumatoid arthritis, inflammatory bowel disease, etc.), decompensated liver cirrhosis, severe kidney disease, epilepsy, cerebrovascular stroke, body mass index above 35 kg/m^2^, pregnant or breastfeeding, and a predictable likelihood of poor compliance.

### Sample Size Considerations

We estimated the sample size based on previous studies that assessed melatonin’s effect on the DPMS measured by the change on the NPS during the CPM-task (Zanette et al., 2014). Accordingly, with six dependent variables and a large effect size (*f* *^2^* = 0.35) to compare melatonin and placebo by multivariate analyses of covariance (MANCOVA), with two predictors in a 1:1 ratio, the estimate indicated a sample size of 32 for a power of 80% and an α of 0.05. Considering possible dropouts, we increased the sample by 12%, and the final sample size comprised of 36 patients (18 per group).

### Randomization and Masking

We used a randomly selected block sizes of 8 and 6. Thirty-six women were allocated to receive melatonin or placebo, an allocation of 1:1. Before the recruitment phase, randomization was computer generated by two investigators uninvolved in the patients’ assessments. Envelopes containing the allocated treatment were prepared, sealed, and numbered sequentially. The envelope was opened following the sequence of numbers registered in the envelope after the participant consented to participate in the trial. Following the conclusion of treatment, we assessed the effectiveness of the blinding protocol by asking patients to guess which treatment they each received (i.e. melatonin, placebo, or unknown).

### Interventions

Patients were instructed to take 20 mg of oral melatonin or placebo daily approximately 1 h before bedtime. Melatonin capsules were produced using crystalline melatonin with a certificate of purity (M-5250, Sigma Chemical, Saint Louis, MO, USA) by a compounding pharmacy. The tablets of melatonin and placebo were physically identical. Assessments to confirm adherence to treatment included: i) Pill counting during the study period. ii) Patient diaries were kept in order to record if they failed to use the medication. iii) Patients were encouraged to remain on melatonin throughout the ten days of treatment.

### Assessments and Instruments

**Figure 2:**
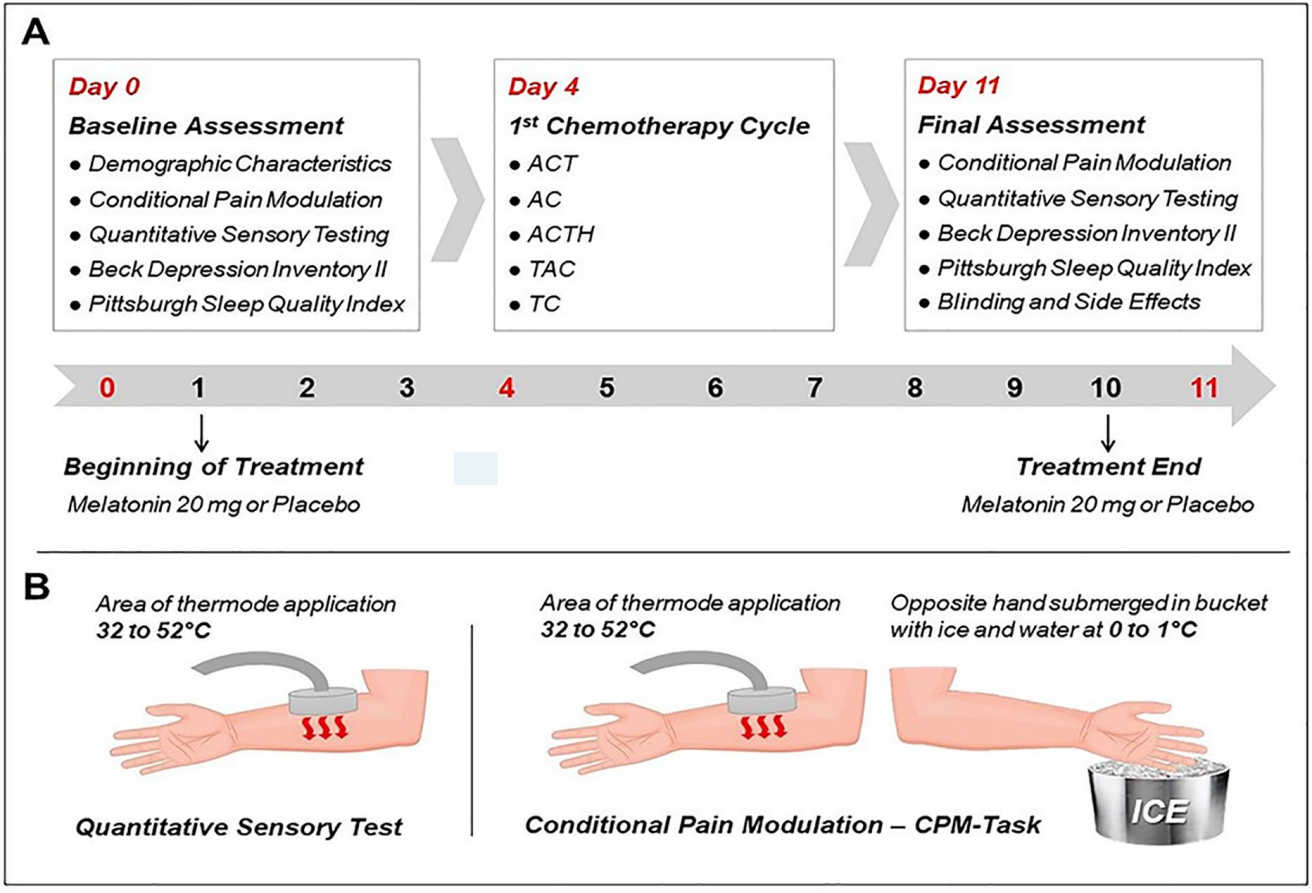
**(A)** Timeline of study. On day 0, the socio-demographic questionnaire, Beck Depression Inventory (BDI-II), and Pittsburgh Sleep Quality Index (PSQI) was applied. Day 0: they begin the treatment; day 4: 1st cycle of chemotherapy; Day 11: BDI-II and PSQI and Questionnaire was applied to assess blinding and side effects. **(B)** Psychophysical measures: Quantitative Sensory Testing (QST) was applied to evaluate the heat pain threshold (HPT), heat pain tolerance (HPTo), and conditioned pain modulation (CPM-test). These measures were assessed on days 0 and 11.

All assessments were conducted by two independently trained research personnel to apply psychophysical pain measurements. The timeline of assessments is presented in **Figure 2**.

#### Outcomes

The treatment effect on primary and secondary outcomes were evaluated by the Δ-value, defined by measurements at treatment end minus values at baseline. Changes in the NPS during the CPM-task assessed the function of the DPMS (primary outcome). The secondary outcomes were the changes produced by treatment in the following measures: HPT, HPTo, BDNF, TrkB, S-100B-protein.

#### Assessment of Primary and Secondary Outcomes

- *QST* was the method utilized in the assessment of HPTs using the method of limits with a computer Peltier-based device thermode (30 × 30 mm) (Schestatsky et al., 2011) that was attached to the skin surface of the ventral portion of the mid-forearm. The initial temperature of the QST is set at 32°C and it increases at a rate of 1°C/s to a maximum of 52°C. The average temperature in °C of three consecutive assessments enough to induce pain comprises the HPT.
- *HPTo* is the temperature induced by the QST to induce the maximum pain tolerated, with a ceiling of 52°C.
- *DPMS* was evaluated by the changes on the NPS ranging from 0 (no pain) to 10 (worst pain imaginable). The CPM was induced by a heterotopic noxious stimulus administered concurrently with a QST enough to produce a pain score of 6/10. The conditioned *pain* modulation (CPM) test was provoked by immersions of the nondominant hand into cold water (0^0^C) for 1 min. During the CPM-task, subjects were asked to rate the pain induced by a pre-defined thermal stimulus to produce a score of 6/10 on the NPS, then 30 seconds later the heterotopic stimulus with cold-water hand immersion was performed. The CPM was defined as the difference between the average pain rating on the NPS before and after cold water immersion.
- *Neuroplasticity state biomarkers* were evaluated using serum levels of BDNF, TrkB, and S100B collected in plastic tubes and centrifuged for 10 min at 4,500 rpm at 4°C in a −80°C freezer for further BDNF and TrkB assays. Serum-mediator concentrations were determined using BDNF (Chemicon CYT306, lower detection limit 7.8 pg/mL; EMD Millipore, Billerica, MA, USA), TrkB (MYBI–MBS9346917, lower detection limit 0.25 ng/ml; MyBiosource, San Diego, CA, USA), S100B (EZHS100B-33 K, Millipore, Missouri, USA, lower detection limit 2.7 pg/mL), and enzyme-linked immunosorbent assay kits in accordance with the manufacturer’s instructions.

#### Clinical Measurements: Depressive Symptoms and Sleep Quality

- *Beck Depression Inventory* is a questionnaire composed of 21 multiple-choice questions with four options each (0–3). The total BDI score ranges from 0–63; higher scores indicate a higher degree of depressive symptoms (Schestatsky et al., 2011).
- *Pittsburgh Sleep Quality Index (PSQI)*. The PSQI is a self-reporting questionnaire that comprises 19-items to assess the quality of sleep and identifies sleep disorders (Warmenhoven et al., 2012). The score ranging from 0 to 21.

#### Other Instruments and Assessments

The patients’ demographic data were assessed using standardized demographic questionnaires. The side effects related to chemotherapy were assessed by the questionnaire of the European Organization for Cancer Research and Treatment validated for the Brazilian population (EORTC QLQ-C30) before and after treatment.

### Statistical Analysis

Inferential tests for demographic and clinical measures, as well as for the psychophysical pain measures and biomarkers of neuroplasticity (i.e. BDNF, TrkB, S100B), were based on independent sample *t*-tests for continuous variables and utilization of the Mann-Whitney non-parametric test. To control the inter-individual variability, existing imbalances between groups, and baseline differences of the DPMS function assessed using the NPS, HPT, HPTo, BDNF, TrkB, and S100B protein, we used the mean differences [delta(Δ)-values (at treatment end minus baseline)] to identifying the real changes in psychophysical and laboratories tests in each patient (Roehrs and Roth, 2005). It recognized that psychophysiological measures show individual reactivity to a stimulus of the same intensity. For example, one individual may be highly reactive into painful stimuli, whereas another shows limited changes receiving the same stimulus. Especially with clinical studies, it may be necessary to not only look at the difference but to acknowledge the starting situations (i.e. adjust for the baseline value). Thus, to control for the inter-individual variability changes in these psychophysical measures and serum markers of neuroplasticity, we compared the effect of treatment on the Δ-values from the baseline to their levels at treatment end. To analyze the treatment effect on all primary and secondary outcomes, we conducted MANCOVA. The dependent variables were the Δ-values of outcome measures [change on NPS (0–10) during the CPM-task, HPT, HPTo, BDNF, TrkB, S-100B]; the treatment group was the factor, and the Δ-values of sleep quality and depressive symptoms were covariates. The rationale to adjust the analysis by these two covariates is supported by evidence of previous studies, which showed that a better functioning of pain inhibition was positively associated with sleep efficiency and with the sleep duration (Lopez et al., 2008; Lopez-Canul et al., 2015a; Innominato and Lim, 2016). Also, a previous study found that the mood can influence the CPM response (Roehrs and Roth, 2005). So, it is plausible to adjust the melatonin effect on the outcomes considering the influence of these potential confounding factors. For this, the treatment effects on outcomes were examined by regression analysis. Also, all analyses were adjusted for multiple comparisons by Bonferroni’s test. The analysis was by the intention-to-treat (ITT) method, we considered all of the randomized patients, and in the case of the dropout, we considered that the patient had a worst-case response in the respective treatment group (melatonin or placebo). For all analyses, we considered a Type I two-sided error (bicaudal) α = 0.025. For statistical analyses, the IBM SPSS Statistics for Windows, Version 20.0 was used (IBM Corp., Armonk, NY, USA).

## Results

### Sociodemographic and Clinical Characteristics

The characteristics of the participants are presented in **Table 1**. Randomization produced balanced groups for most of the characteristics, except in years of school. In the melatonin and placebo group, 13 (54.2%) vs. 11 (45.8%) assumed to have received melatonin, respectively. In the melatonin and placebo group, 4 (44.4%) vs. 5 (55.6%) assumed that they received placebo, respectively. Two in the melatonin group and one in the placebo group assumed that their treatment was unknown (P = 0.69). Regarding the severity of the adverse effect scores, according to EORTC the median and interquartile (Q_25–75_) were observed to be at 10 (Q_25–75_ = 2; 20) vs. 9 (Q_25–75_ = 0; 24), P = 0.35, in the melatonin and placebo group, respectively. We observed that melatonin treatment reduced the severity of adverse effects as the median and interquartile (Q_25–75_) was 7 (Q_25–75_ = 2; 19) vs. 12.5 (Q_25–75_ = 3; 25), *P* = 0.01, in the melatonin and placebo group, respectively.

**Table 1:** Baseline demographic and clinical characteristics according to treatment group. Data are presented as mean standard deviation (SD) (n = 36).

Variables	Melatonin (n = 18)	Placebo (n = 18)	*P*-value
Age (years)	54.24 (10.59)	54.11 (9.15)	0.97
Formal education (years)	9.29 (4.04)	6.94 (2.57)	0.08†
Body mass index (kg/m^2^)	28.0 (6.14)	29.94 (5.70)	0.25†
Visual analog scale (0–100)	50 (20.00)	50 (16.48)	0.80
Brain-derived neurotrophic factor (ng/mL)	42.92 (17.54)	42.24 (23.95)	0.92
Tropomyosin receptor kinase B (ng/mL)	0.48 (0.25)	0.47 (0.50)	0.49
Protein S100B (pg/mL)	38.16 (12.42)	32.37 (8.93)	0.21
Pittsburgh Sleep Quality Index	8.24 (3.97)	8.44 (2.83)	0.86
Beck Depression Inventory II	11.41 (7.73)	10.83 (5.11)	0.79
**Chronic disease**			
Hypertension	7 (38.9%)/11 (61.1%)	8 (44.4%)/10 (55.6%)	
Hypothyroidism	3 (16.7%)/15 (83.3%)	1 (5.6%)/17 (94.4%)	
Diabetes mellitus	1 (5.6%)/17 (94.4%)	1 (5.6%)/17 (94.4%)	
Asthma	1 (5.6%)/17 (94.4%)	1 (5.6%)/17 (94.4%)	
Psychotropic medication (yes/no)*			
Number of psychotropic medications	8/18 (44.44%)	9/18 (50%)	
Selective serotonin reuptake inhibitors	3 (16.7%)/15 (83.3%)	3 (16.7%)/15 (83.3%)	
Tricyclics	1 (5.6%)/17 (94.4%)	2 (11.1%)/16 (88.9%)	
Benzodiazepines	3 (16.7%)/15 (83.3%)	4 (22.2%)/14 (77.8%)	
Antipsychotics	1 (5.6%)/17 (94.4%)	::::	
**Chemotherapy regimens (yes/no)**			
ACT (doxorubicin plus cyclophosphamide followed by weekly paclitaxel)^1^	9 (50%)/9 (50%)	9 (50%)/9 (50%)	
AC (doxorubicin plus cyclophosphamide)^1^	5 (27.8%)/13 (72.2%)	2 (11.1%)/16 (88.9%)	
ACTH (doxorubicin plus cyclophosphamide followed by paclitaxel plus trastuzumab)^1^	2 (11.1%)/16 (88.9%)	3 (16.7%)/(83.3%)	
TAC (docetaxel, doxorubicin, and cyclophosphamide)^2^	1 (5.6%)/17 (94.4%)	2 (11.1%)/16 (88.9%)	
TC (docetaxel plus cyclophosphamide)^2^	1 (5.6%)/17 (94.4%)	2 (11.1%)/16 (88.9%)	

^†^Mann-Whitney non-parametric test was used. Independent t-tests were applied to all other measures.

*Three patients use more than one psychotropic medication.

Prophylaxis for infusion reactions:

^1^Dexamethasone 20 mg IV 30 min before drug administration.

^2^Dexamethasone 8 mg orally every 12 h starting one day prior to docetaxel administration.

### Primary and Secondary Outcomes

#### Univariate Analysis of the Primary Outcome to Compare the Treatment Group Effect on the NPS (0–10) During the CPM Task

**Figure 3:**
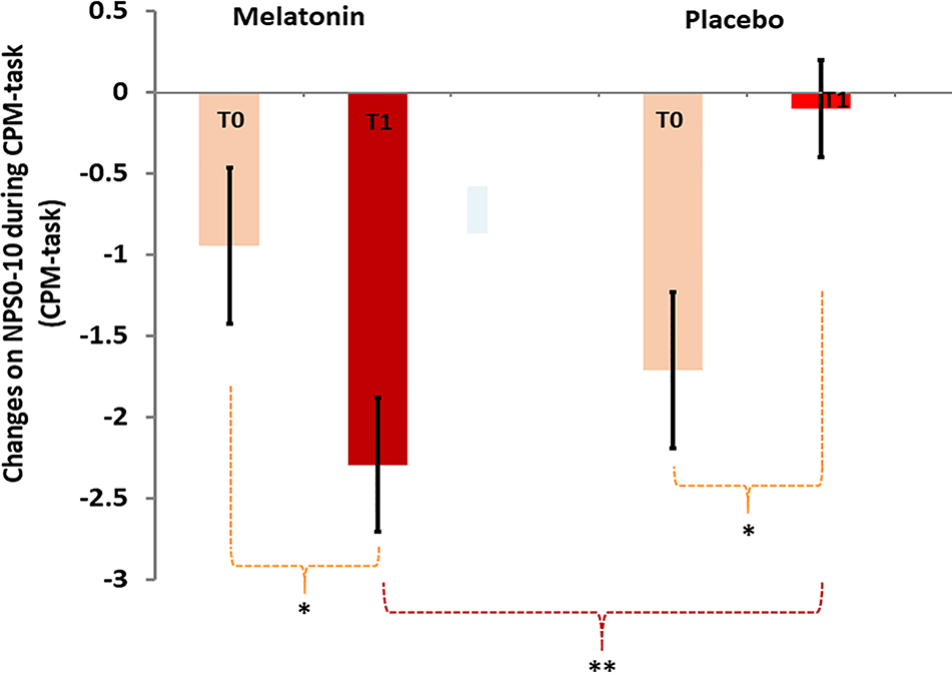
Change on the NPS (0–10) during the CPM-task at baseline (T0) and treatment end (T1). Error bars indicate standard error of the mean (SEM). Asterisks (*) positioned above symbols mean difference within and (**) mean difference between groups.

The efficiency of the DPMS assessed by the change on the NPS during the CPM-task increased 43.5% from T0 to T1 in the melatonin group, whereas it decreased 93% in the placebo group [*t* = -4.14, df = 33.57; *P* < 0.001]. The mean on the NPS during the CPM-task at T0, T1 and the Δ-value is presented in **Table 2** and **Figure 3**.

**Table 2:** Pain psychophysical measures and serum markers of neuroplasticity state at treatment end according to melatonin or placebo groups.

	Placebo (n = 18)		Melatonin		***P*** -value*
	**Mean (SD)**	**Δ-value**	**Mean (SD)**	**Δ-value**	
***Primary outcome***					
Conditional pain modulation: change on NPS (0–10) during CPM-task					
Baseline	−1.81 (1.67)	−1.91(1.60)	−0.94 (1.61)	−3.25 (1.61)	<0.001
End treatment	−0.10 (1.52)	−2.29 (1.61)			
***Secondary outcomes***					
Heat pain threshold					
Baseline	40.99 (2.52)	−3.05 (2.74)	38.47 (2.58)	2.46 (1.98)	<0.001
End treatment	37.94 (2.99)	40.99 (1.92)			
Heat pain tolerance					
Baseline	49.78 (2.62)	−1.18 (1.95)	49.01 (2.66)	1.32 (2.07)	0.001
End treatment	48.59 (2.96)	50.33 (1.79)			
Brain-derived neurotrophic factor (BDNF)					
Baseline	40.88 (23.78)	1.87 (7.17)	41.65 (17.72)	−20.44 (17.17)	<0.001
End treatment	42.76 (17.75)	21.31 (7.18)			
Tropomyosin kinase receptor b (TrkB)					
Baseline	0.47 (0.50)	0.46 (0.17)	0.56 (0.39)	−0.15 (0.18)	0.003
End treatment	0.52 (0.46)	0.41 (0.37)			
S100 calcium binding protein B protein (S100B)					
Baseline	33.21 (9.25)	2.89 (11.18)	38.17 (12.42)	−11.16 (9.75)	<0.001
End treatment	36.11 (12.19)	26.96 (8.45)			
Pittsburgh Sleep Quality Assessment					
Baseline	8.44 (2.83)	2.83 (2.31)	8.24 (3.98)	−3.18 (2.01)	<0.001
End treatment	11.06 (3.35)	5.06 (3.34)			
Beck Depression Inventory II					
Baseline	10.83 (5.11)	3.72 (5.21)	11.41 (7.73)	−4.71 (5.83);	<0.001
End treatment	14.56 (7.76)	6.41 (4.57)			

*Correspond to comparisons of Δ-value by the t-test for independent sample. Data are presented as the mean prior, posttreatment and Δ-value (post minus prior) and standard deviation (SD) (n = 36).

#### Multivariate Analysis of Primary and Secondary Outcomes to Compare the Treatment Group Effect on the Psychophysical Pain Measures Considering Melatonin’s Effect on the Neuroplasticity State and Sleep Quality

The MANCOVA analysis to compare between groups of mean differences [delta(Δ)-values of mean difference averages of measures (at treatment end minus the baseline means)] with the adjustment for multiple comparisons is presented in **Table 3**. The MANCOVA analysis revealed a significant main effect of treatment; Pillai’s Trace’s F (Kaila-Kangas et al., 2006; Warmenhoven et al., 2012) = 5.11; *P* < 0.001; η²partial = 0.56 (**Table 3A**). The Δ-mean (SD) on the NPS (0–10) during the CPM-task of the placebo was −1.91 [−1.81 (1.67) vs. −0.1 (1.61)], and in the melatonin group was −3.5 [−0.94 (1.61) vs. −2.29 (1.61)], and the mean difference (md) between treatment groups were 1.59 [(95% CI, 0.50 to 2.68), η²partial = 0.60 indicating a large effect size (see **Table 3A**). It confirmed that melatonin’s effect optimizes the DPMS supported by the change on the NPS during the CPM-task. Also, it increased the HPT and HPTo, while reducing the serum levels of BDNF, TrkB, and S-100B. In **Table 3B**, the coefficients of the linear regression analysis of MANCOVA are presented. The result showed that the Δ-PSQI was negatively correlated with the Δ-value of changes on NPS (0–10) during the CPM-task (Standardized Beta = −0.37; *t* = −2.20, *P* = 0.03, η²partial = 0.13). It is important to remember that a higher change on the Δ-PSQI means a better effect of melatonin on sleep quality, while a larger change on the NPS during the CPM-task indicates that the heterotopic stimulus was more effective. Hence, the difference in the NPS (PPT1 minus PPT0) produced a higher negative value. Thus, this explains the coherence of this negative correlation. The interaction analysis showed that melatonin’s effect on the DPMS was not related to its impact on the improvement of sleep quality (Standardized Beta = 0.20, *t* = 0.78 P = 0.44).

**Table 3:** MANCOVA model to compare the treatment effect in the Δ-value of psychophysical pain measures and the neuroplasticity state considering melatonin’s effect, depressive symptoms and sleep quality (n = 36).

*(A) Main effects*						
*Corrected model*	*Type III sum of squares*	*df*	*Mean square*	*F*	*P-value*	*η²* *_partial_*
**Dependent variables**						
Δ- Changes on NPS0–10 during CPM-task	102.47^a^	4	25.62	10.99	<0.01	0.60
Δ- Heat pain threshold	285.03^b^	4	71.26	13.18	<0.01	0.65
Δ-Heat pain tolerance	79.17^c^	4	19.79	5.56	<0.01	0.43
Δ-Brain-derived neurotrophic factor (BDNF)	5,176.77^d^	4	1,294.19	7.89	<0.01	0.52
Δ- Tropomyosin receptor kinase B (TrkB)	0.62^e^	4	0.16	6.13	<0.01	0.46
Δ- S100 calcium binding protein B (S-100B)	1,871.64^f^	4	467.91	3.88	0.01	0.35
**Intercept**						
Δ- Changes on NPS0–10 during CPM-task	4.40	1	4.40	1.89	0.18	0.06
Δ- Heat pain threshold	1.18	1	1.18	0.22	0.65	0.01
Δ-Heat pain tolerance	1.47	1	1.47	0.41	0.53	0.01
Δ-Brain-derived neurotrophic factor (BDNF)	2,133.52	1	2,133.52	13.01	<0.01	0.31
Δ- Tropomyosin receptor kinase B (TrkB)	0.16	1	0.16	6.37	0.02	0.18
Δ- S100 calcium binding protein B (S-100B)	405.62	1	405.61	3.36	0.08	0.10
**Treatment group**						
Δ- Changes on NPS0–10 during CPM-task	59.85	1	59.85	25.69	<0.01	0.47
Δ- Heat pain threshold	154.14	1	154.14	28.50	<0.01	0.49
Δ-Heat pain tolerance	60.56	1	60.56	17.01	<0.01	0.37
Δ-Brain-derived neurotrophic factor (BDNF)	2,375.01	1	2,375.01	14.48	<0.01	0.33
Δ- Tropomyosin receptor kinase B (TrkB)	0.45	1	0.46	18.01	<0.01	0.38
Δ- S100 calcium binding protein B (S-100B)	703.52	1	703.52	5.83	0.02	0.17
**Δ-Pittsburgh Sleep Quality Index**						
Δ- Changes on NPS0–10 during CPM-task	9.95	1	9.95	4.27	0.048	0.13
Δ- Heat pain threshold	24.57	1	24.57	4.54	0.042	0.14
Δ-Heat pain tolerance	14.37	1	14.37	4.04	0.054	0.12
Δ-Brain-derived neurotrophic factor (BDNF)	208.11	1	208.11	1.27	0.269	0.04
Δ- Tropomyosin receptor kinase B (TrkB)	0.11	1	0.11	4.32	0.047	0.13
Δ- S100 calcium binding protein B (S-100B)	8.18	1	8.175	0.08	0.796	<0.01
**Δ-Beck Depression Inventory-II**						
Δ- Changes on NPS0–10 during	0.32	1	0.32	0.14	0.71	<0.01
Δ- Heat pain threshold	0.03	1	0.03	0.01	0.94	<0.01
Δ-Heat pain tolerance	4.79	1	4.79	1.35	0.26	0.04
Δ-Brain-derived neurotrophic factor (BDNF)	2.47	1	2.47	0.06	0.90	<0.01
Δ- Tropomyosin receptor kinase B (TrkB)	0.04	1	0.04	1.59	0.22	0.05
Δ- S100 calcium binding protein B (S-100B)	14.71	1	14.71	0.12	0.73	<0.01
Grupo * Δ-Pittsburgh Sleep Quality Index						
Δ- Changes on NPS0–10 during CPM-task	1.42	1	1.41	0.61	0.44	0.02
Δ- Heat pain threshold	0.13	1	0.11	0.02	0.89	<0.01
Δ-Heat pain tolerance	3.86	1	3.86	1.09	0.31	0.04
Δ-Brain-derived neurotrophic factor (BDNF)	419.34	1	419.34	2.56	0.12	0.08
Δ- Tropomyosin receptor kinase B (TrkB)	0.08	1	0.08	3.00	0.09	0.09
Δ- S100 calcium binding protein B (S-100B)	71.13	1	71.13	0.59	0.45	0.02
***(B) Coefficients***						
		***Beta***	***SEM***	***t***	***P-value***	***CI 95%***
**Dependent variable: Δ-changes on NPS (0–10) during the CPM-task**						
Treatment group	Melatonin	−4.97	0.98	−5.07	0.00*	(−6.98 to −2.97)
	Placebo	0^reference^				
Δ-Pittsburgh Sleep Quality Index		−0.37	0.17	−2.20	0.03*	(−0.70 to −0.03)
Δ-Beck Depression Inventory-II		−0.02	0.05	−0.37	0.71	(−0.12 to 0.09)
*Interaction*						
Melatonin* Δ-Pittsburgh Sleep Quality Index		0.20	0.27	0.78	0.44	(−0.33 to 0.75)
Placebo* Δ-Pittsburgh Sleep Quality Index		0^reference^				
**Dependent variable: Δ-heat pain threshold**						
Treatment group	Melatonin	7.981	1.49	5.34	0.00*	(4.92 to 11.03)
	Placebo	0^reference^				
Δ-Pittsburgh Sleep Quality Index		0.44	0.25	1.75	0.09	(−0.07 to 0.96)
Δ-Beck Depression Inventory-II		−0.006	0.08	−0.08	0.94	(−0.16 to 0.15)
*Interaction*						
Melatonin* Δ-Pittsburgh Sleep Quality Index		−0.06	0.40	−0.14	0.88	(−0.88 to 0.77)
Placebo* Δ-Pittsburgh Sleep Quality Index		0^reference^				
**Dependent variable: Δ-heat pain tolerance**						
Treatment group	Melatonin	5.00	1.21	4.12	0.00*	(2.52 to 7.48)
	Placebo	0^reference^				
Δ-Pittsburgh Sleep Quality Index		0.49	0.20	2.38	0.02*	(0.06 to 0.90)
Δ-Beck Depression Inventory-II		0.07	0.06	1.16	0.25	(−0.006 to 0.20)
*Interaction*						
Melatonin* Δ-Pittsburgh Sleep Quality Index		−0.34	0.33	−1.04	0.30	(−1.0 to 0.32)
Placebo * Δ-Pittsburgh Sleep Quality Index		0^reference^				
**Dependent variable: Δ-Brain-derived neurotrophic factor (BDNF)**						
Treatment group	Melatonin	−31.33	8.23	−3.80	0.00*	(−48.16 to −14.49)
	Placebo	0^reference^				
Δ-Pittsburgh Sleep Quality Index		0.58	1.39	0.42	0.68	(−2.26 to 3.41)
Δ-Beck Depression Inventory-II		−0.05	0.42	−0.12	0.90	(−0.93 to 0.82)
*Interaction*						
Melatonin* Δ-Pittsburgh Sleep Quality Index		−3.55	2.22	−1.56	0.12	(−8.09 to 0.90)
Placebo* Δ-Pittsburgh Sleep Quality Index		0^reference^				
**Dependent variable: Δ- Tropomyosin receptor kinase B (TrkB)**						
Treatment group	Melatonin	−0.43	0.102	−4.24	0.00*	(−0.64 to −0.23)
	Placebo	0^reference^				
Δ-Pittsburgh Sleep Quality Index		−0.004	0.017	0.20	0.84	(−0.04 to 0.03)
Δ-Beck Depression Inventory-II		−0.007	0.005	−1.26	0.21	(−0.02 to 0.004)
*Interaction*						
Melatonin* Δ-Pittsburgh Sleep Quality Index		−0.05	0.03	−1.73	0.09	(−0.10 to 0.009)
Placebo* Δ-Pittsburgh Sleep Quality Index		0^reference^				
**Dependent variable: Δ- S100 calcium binding protein B (S-100B)**						
Treatment group	Melatonin	−17.05	7.06	−2.41	0.02*	(−31.49 to −2.61)
	Placebo	0^reference^				
Δ-Pittsburgh Sleep Quality Index		0.49	1.18	0.41	0.68	(−1.94 to 2.92)
Δ-Beck Depression Inventory-II		−0.13	0.36	−0.35	0.73	(−0.88 to 0.62)
*Interaction*						
Melatonin* Δ-Pittsburgh Sleep Quality Index		−1.46	1.90	−0.77	0.45	(−5.35 to 2.43)
Placebo* Δ-Pittsburgh Sleep Quality Index		0^reference^				

R^2^ = 0.603 (adjusted R^2^ = 0.548)^a^

R^2^ = 0.645 (adjusted R^2^ = 0.596)^b^

R^2^ = 0.434 (adjusted R^2^ = 0.356)^c^

R^2^ = 0.521 (adjusted R^2^ = 0.455)^d^

R^2^ = 0.458 (adjusted R^2^ = 0.384)^e^

R^2^ = 0.349 (adjusted R^2^ = 0.259)^f^

The Δ-values of means (Δ-means) of each group (mean at treatment end minus mean before treatment) and the mean difference between the melatonin vs. placebo group, with their respective confidence interval (CI; 95%) compared using MANCOVA and adjusted for multiple comparisons showed that the melatonin reduced the serum levels of the neuroplasticity markers. Melatonin’s effect reduced the (Δ)-means of the serum neuroplasticity marker in placebo vs. melatonin The Δ-BDNF was 1.87 (7.17) vs. −20.44 (17.17), respectively, and the md = 22.31 [(95% CI = 13.40 to 31.22)]; TrKB md = 0.61 [0.46 (0.17) vs. −0.15 (0.18); 95% CI = 0.49 to 0.73)] and S00B-protein md = −8.27[(2.89 (11.18) vs. −11.16 (9.75); 95% CI = -15.38 to −1.16)]. However, it was observed that melatonin compared to placebo increased both the HPT [Δ-means (SD) [2.46 (1.98) vs. −3.06 (2.74); md = −5.52, 95% CI (−7.14 to −3.90)] and HPTo [[Δ-means (SD) 1.32 (2.07) vs. −1.18 (1.95); md = -2.5, 95% CI = −3.86 to −1.14)], respectively.

## Discussion

These findings confirm the benefits of melatonin compared to placebo prior to and during the first cycle of ACBC by counteracting the neurotoxic effects on the inhibitory function of the DPMS evaluated by the change on the NPS during the CPM-task. Melatonin also increased the HPT and HPTo, while reducing the serum levels of the neuronal and astrocyte neuroplastic markers (i.e. BDNF, TrkB, and S100B-protein). The analysis showed that the effect of melatonin on the DPMS and the neuroplasticity state was not related to its impact on sleep quality.

The novelty of this study was to reveal that melatonin may counteract processes related to ACBC that produces dysfunction in the inhibitory DPMS and in the neuroplastic state. These findings corroborate our previous results of melatonin’s effect on the DPMS in fibromyalgia patients (Zanette et al., 2014) and provides mechanistic support to explain the high prevalence of pain claims in patients receiving ACBC. Also, these findings extend evidence as to how melatonin’s effects on improving the inhibitory DPMS and changes in the neuroplasticity state are independent of its impact related to improving sleep quality. Thereby, these findings show that the influence of melatonin on the neural plasticity field can induce improvement in clinical outcomes related to pain and sleep quality.

Although extensive literature supports the relationship between poor sleep quality and chronic pain, this relationship has been comprehended as a vicious cycle (Smith et al., 2007). According to pre-clinical studies, sleep deprivation induces a synaptic instability in spinal cord neurons (Lopez et al., 2008), which may produce the imbalance between the excitability and inhibitory mechanisms. Considering that the melatonin has the potential to improve sleep quality (Innominato and Lim, 2016), we conducted an interaction analysis between the intervention group and sleep quality. This analysis allows exploring if the improvement in sleep quality has mediated the melatonin effect on pain measures. The interaction analysis revealed that the melatonin effect on pain perception and in the descending pain inhibitory system was independent of its impact on sleep quality and that the effect of melatonin on pain involved other mechanisms: (i) According to pre-clinical studies, in rats, the administration of selective MT2 receptor partial agonist UCM924 in the periaqueductal gray (PAG) may induce an antiallodynic effect in two models of neuropathic pain. This effect was entirely blocked by the MT2 receptor antagonist 4-phenyl-2-propionamidotetralin (4P-PDOT) (Lopez-Canul et al., 2015a). Altogether, these findings demonstrate the involvement of MT2 receptor analgesic properties through modulation of a brainstem descending antinociceptive pathways. In another study, Lopez-Canul et al. (2015b) showed that the antinociceptive properties of UCM765 and UCM924 in acute and inflammatory pain models and corroborate the concept that MT2 melatonin receptor may be a novel target for analgesic effect. (ii) Although in the current study the melatonin effect on pain is likely mediated by mechanisms that are not directly related to sleep quality, we cannot exclude that the improvement in sleep efficiency and the sleep duration had affected the pain inhibition by other alternative mechanisms. This is, plausible since the relationship between sleep quality and the inhibitory function of DPMS has been found in different pain conditions for example in temporomandibular joint disorders (Vidor et al., 2013), rheumatoid arthritis (Purabdollah et al., 2017), and fibromyalgia (Brietzke et al., 2019). It is noteworthy that the periaqueductal gray matter is a fundamental DPMS structure (Leknes et al., 2013) and that the function of DPMS has been correlated with sleep effectiveness (Carvalho et al., 2019). (iii) According to pre-clinical studies, the pain inhibitory system at the periaqueductal gray depends on endogenous opioids and noradrenergic projections from the locus coeruleus (Villapol et al., 2011; Jing et al., 2014; Reiter et al., 2014; Serikov and Lyashev, 2015), in such a way, that the descending pain inhibitory system and sleep share neurobiological mechanisms. (iv) Aligned with this perspective to comprehend the mechanisms related to sleep and the role of DPMS, an earlier study using functional magnetic resonance imaging (fMRI) revealed that FM patients compared to healthy controls showed a deactivation the rostral anterior cingulate cortex (rACC) (Ichesco et al., 2016). This study suggests that the dysfunctional brainstem structures involved in pain inhibition are also involved either in sleep regulation. In the same way, another study revealed that sleep disturbances seem to engender the impairment of DPMS (Carvalho et al., 2019).

This study indicates that melatonin’s effect improved of the DPMS function, as well as reduced serum levels of BDNF. These results are in line with evidence from previous studies on chronic pain using melatonin, as serum BDNF was found to be reduced in patients with endometriosis (Schwertner et al., 2013), while in fibromyalgia the DPMS was improved (Zanette et al., 2014). Accordingly, mechanistic studies indicate that melatonin up-regulates gene expression of serum BDNF while pharmacological studies showed a direct role of TrkB signaling in the development of neuropathic pain (de Zanette et al., 2014). Thus, these results support the notion that melatonin contra-regulates the disruption in the BDNF–TrkB signaling induced by ACBC, which is crucial for the development, plasticity, and remodeling of neuronal circuits (Ren and Dubner, 2007). In this way, preclinical studies have demonstrated that TrkB inhibition has significant favorable effects in animal models regarding neuropathic pain, depression, cancer, and addictive behavior (Ghilardi et al., 2010; Yu and Chen, 2011; Zhou et al., 2017).

Other additional mechanisms to explain the neuroprotective impacts of melatonin’s ability to counteract the neurotoxicity of ACBC include anti-apoptotic, anti-inﬂammatory, and antioxidant effects (Zhang and Zhang, 2014; Manchester et al., 2015). Indeed, it is possible that the multiplicity of melatonin’s properties reduces transient apoptosis in the brain induced by chemotherapy, as well as reducing neurotoxicity by reversing the microvasculature changes or cytokine activity responsible for diminishing neurogenesis and neuroplasticity. Additionally, a decrease of serum S100B concentration in our results supports that melatonin provides a modulatory effect on astrocytic activity (Gonçalves et al., 2008). In this way, it is also congruent with the function of serum S100B, a neurotrophic factor that may increase neural survival, neurite extension, and suppression of glial reactivity (Donato R et al., 2013). However, S100B-protein is toxic at very high concentrations (Donato, 2001). Present data further supports, due to its ability to surpass biological barriers such as the blood-brain barrier (BBB) (Marchi et al., 2003), that S100B inhibits inﬂammatory pathways that would cause brain damage. According to most pre-clinical studies on neuronal damage, melatonin is often given within a 1–20 mg/kg dose range (Ananth et al., 2003; Lee et al., 2005; Yon et al., 2006), while at doses higher than 5 mg/kg it provides maximum neuroprotection in ischemic stroke models (Macleod et al., 2005). However, we cannot transpose the doses used in pre-clinical studies to humans without evaluating its effects on humans due to pharmacokinetic differences.

Similarly, BDNF can cross the BBB bidirectionally, therefore a substantial portion of its serum levels originate from neuronal and glial cells, reﬂecting its neuronal concentration. Thus, BDNF concentrations found in the brain have been correlated with its serum concentrations (Karege et al., 2002), suggesting that the neuroprotective effects of melatonin may involve a reduced release of BDNF. Similarly, in a study examining subjects with depression, melatonin safeguarded hippocampal neurons from damage *via* BDNF or glial cell-derived neurotrophic factor activation (Oglodek et al., 2016). Contrarily, the S100B astroglial protein released in response to neuronal injury, exerts neurotrophic effects on neurons and glial cells (Sorci et al., 2010). As an astrocytic marker, S100B can be easily detected in the serum (Thelin et al., 2017), and an increase or decrease in levels has been observed in multiple known brain disorders. Although serum S100B may be elevated in acute neuronal damage, S100B levels were decreased in patients with underlying neurodegenerative disease (Chaves et al., 2010) and also observed in patients who carry lower pain thresholds diagnosed with fibromyalgia (Zanette et al., 2014).

Several concerns regarding to our study must be addressed: First, the homogeneity of our sample gives rise to the issue of external validity as it is methodologically advantageous to answer the question of this study. Second, the awareness of group allocation assessment (either active or placebo) demonstrated that blinding was guaranteed, since the rate of patients of the melatonin group believed to have received placebo, or vice-versa, was very similar. Further, our objective surrogate biomarkers and psychological measurements are less susceptible to bias. Hence the unblinding issue is unlikely to have affected our conclusions. Third, a positive effect of melatonin was to reduce the adverse effects due to ACBC. Likewise, 20 milligrams of melatonin or lower as an adjuvant to cancer care with and without chemotherapy reduced asthenia, leucopenia, nausea and vomiting, hypotension, and thrombocytopenia (Seely et al., 2012). However, it was reported that melatonin presents considerable variability in serum levels of 10 to 100 times with a bioavailability ranging from 10% to 56% among healthy subjects receiving the same dose (Brzezinski, 1997). This variability in the pharmacokinetics of melatonin can explain at least part of the mixed results found in the melatonin effect among studies (Rasmussen et al., 2015). Fourth, although earlier research suggests that either chemotherapy regimen (Boland et al., 2014) or psychotropic medications can influence pain processing (Ong et al., 2019) in the present study, the distribution of these variables was very similar between groups indicating that the randomization worked (**Table 1**), whereas it is improbable that a minimal difference in these factors changes the directions of our conclusions.

In conclusion, these results suggest that oral melatonin together with first ACBC counteracts the dysfunction in the inhibitory DPMS and improves pain perception measures. Also, it shows that changes in the neuroplasticity state mediate the impact of melatonin on pain.

## Data Availability Statement

The datasets generated for this study are available on request to the corresponding author.

## Ethics Statement

This randomized, double-blinded, placebo-controlled trial was approved by the Institutional Review Board of Hospital de Clínicas of Porto Alegre (IRB HCPA/Approval number: 14-0701). We obtained oral and written informed consent from all patients before participating in this study. The identified data related to interventions and primary outcomes will be available upon request to interested to WC (wcaumo@hcpa.edu.br) with no time restriction.

## Author Contributions

AP conceived the study and participated in its design, coordination, sequence alignment, and drafting of the manuscript. AS, AZ participated in the design of the study, completion of the statistical analysis, and drafting of the manuscript. IT participated in the sequence alignment and drafting of the manuscript. JC, FS participated in the sequence alignment. VS participated in the design of the study and completion of the statistical analysis. FF participated in the design of the study and coordination, as well as with help drafting the manuscript. WC conceived the study and participated in its design, coordination, sequence alignment, and drafting of the manuscript.

## Funding

The present research was funded *via* the following Brazilian agencies: (i) Committee for the Development of Higher Education Personnel (CAPES PNPD [Grant to 2015)]. (ii) National Council for Scientific and Technological Development [(CNPq (Research grant: IT 302345/2011-6 and WC 30 1256/ 201 3-6)]. (iii) Hospital de Clínicas de Porto Alegre (HCPA) Research Fund (FIPE: 14-0701). (iv) Foundation for Research of the State of Rio Grande do Sul (FAPERGS). (v) Studies and Projects Financing Agency (FINEP: 1245/13).

## Conflict of Interest

The authors declare that the research was conducted in the absence of any commercial or financial relationships that could be construed as a potential conflict of interest.
